# From Capture to Inhibition: How does Irrelevant Information Influence Visual Search? Evidence from a Spatial Cuing Paradigm

**DOI:** 10.3389/fnhum.2016.00232

**Published:** 2016-05-20

**Authors:** Christine Mertes, Edmund Wascher, Daniel Schneider

**Affiliations:** Leibniz Research Centre for Working Environment and Human Factors, Technische Universität DortmundDortmund, Germany

**Keywords:** visual selective attention, N2pc, cognitive control, distractor positivity, CDA

## Abstract

Even though information is spatially and temporally irrelevant, it can influence the processing of subsequent information. The present study used a spatial cuing paradigm to investigate the origins of this persisting influence by means of event-related potentials (ERPs) of the EEG. An irrelevant color cue that was either contingent (color search) or non-contingent (shape search) on attentional sets was presented prior to a target array with different stimulus-onset asynchronies (SOA; 200, 400, 800 ms). Behavioral results indicated that color cues captured attention only when they shared target-defining properties. These same-location effects persisted over time but were pronounced when cue and target array were presented in close succession. N2 posterior contralateral (N2pc) showed that the color cue generally drew attention, but was strongest in the contingent condition. A subsequently emerging contralateral posterior positivity referred to the irrelevant cue (i.e., distractor positivity, Pd) was unaffected by the attentional set and therefore interpreted as an inhibitory process required to enable a re-direction of the attentional focus. Contralateral delay activity (CDA) was only observable in the contingent condition, indicating the transfer of spatial information into working memory and thus providing an explanation for the same-location effect for longer SOAs. Inhibition of this irrelevant information was reflected by a second contralateral positivity triggered through target presentation. The results suggest that distracting information is actively maintained when it resembles a sought-after object. However, two independent attentional processes are at work to compensate for attentional distraction: the timely inhibition of attentional capture and the active inhibition of mental representation of irrelevant information.

## Introduction

Visual attention helps us to select information that is relevant for the pursuit of our behavioral goals. The interaction between bottom-up influence and top-down feedback determines which stimuli gain access to limited processing resources and are represented in higher cognitive areas (Desimone and Duncan, 1995). Top-down mechanisms bias the allocation of attention in favor of relevant information. Bottom-up mechanisms select those stimuli that are physically the most striking ones (Reynolds and Chelazzi, 2004). The selected information is maintained in visual working memory and these representations are provided for further cognitive operations that enable an appropriate and flexible adaption of our behavior (Baddeley, 1996, 2010; Schneider and Wascher, 2013; Schneider et al., 2014).

Although attention can be voluntarily directed towards relevant information, it happens to be captured by irrelevant objects in our visual surrounding. These involuntary shifts of attention are initiated by the same mechanisms that help us to direct our attention towards the most relevant information. How does the attentional system exactly deal with irrelevant information and which different steps are engaged to guarantee the re-orienting to the sought-after objects? The signal suppression hypothesis of controlled attentional capture (Sawaki and Luck, 2010, 2011) is a hybrid account that integrates the influence of bottom-up and top-down mechanisms during attentional capture by distracting information in our visual surrounding. It postulates that distractors automatically generate an attend-to-me priority signal, but that active suppression mechanisms are engaged to act against the deployment of attention to irrelevant information. This priority signal could be evoked by salient stimuli as well as by stimuli that match the representations held in visual working memory (Sawaki and Luck, 2011; Schneider et al., 2012).

In a recent study, Sawaki and Luck (2013) examined the processes occurring when attention is involuntarily captured by distractors. Participants performed a spatial cuing task in which a cue display that contained an irrelevant, spatially non-predictive feature singleton cue was followed by a target display. That kind of experimental design was often used to show that attentional capture is contingent on attentional control sets (contingent involuntary orienting hypothesis; e.g., Folk et al., 1992, 1994; Eimer and Kiss, 2008). Like in previous studies, the participants’ responses were faster when the target and the preceding cue shared the same location, which indicates that attention was already drawn to the location of the cue and so facilitates the processing of the object subsequently presented at that location. In accordance with the contingent involuntary orienting hypothesis, these spatial cuing effects are only found when cue features match the current attentional control set.

Furthermore, to enable a more precise look at the time course of attentional capture, Sawaki and Luck measured event-related potentials (ERPs) evoked by the cue display. The authors observed an N2 posterior contralateral (N2pc; Luck and Hillyard, 1994) to the singleton cue, a negative-going deflection over posterior scalp electrodes contralateral to the attended stimulus. It starts at about 200 ms after the stimulus onset and provides evidence that participants directed their attention towards the actually irrelevant cue when it matches working memory templates (Eimer and Kiss, 2008). Subsequently to N2pc, a component labeled distractor positivity (Pd; Hickey et al., 2009; Hilimire et al., 2012) occurred. Pd indexes active attentional inhibition (Hickey et al., 2009; Sawaki and Luck, 2011, 2013; Kiss et al., 2012; Sawaki et al., 2012) and arises for salient distractors just as it does for those that match an activated control set (Sawaki et al., 2012). This component shows up as a more positive deflection contralateral to the location of the to be suppressed stimulus. Its onset and time course varies with the experimental design but occurs at about 200 ms after stimulus onset subsequently or simultaneously to N2pc. In the context of contingent attentional capture Sawaki and Luck (2013) interpreted the occurrence of Pd as an active suppression process of the singleton cue’s location that enables the rapid re-orienting to the target in the search array. However, the suppressive mechanism indexed by Pd component seems not to be entirely completed (Lien et al., 2008). Faster responses were reported for targets that were preceded by cues in the same quadrant. So, even if attention could be rapidly withdrawn from the cued location, it was initially deployed to this location. Therefore it must be assumed that some information of the cue was maintained until the presentation of the target. This corresponds to findings recently reported by Schneider et al. (2015, 2016). They observed an increase in alpha power towards encoded but no longer needed information in a retroactive-cuing paradigm, reflecting the inhibition of these irrelevant signals or the absence of active retention of the irrelevant information in working memory. Nevertheless, targets resembling the irrelevant information triggered a more time demanding comparison process than stimuli not previously encoded into working memory. Comparable to the findings of Sawaki and Luck (2013), the inhibitory processes engaged by the attentional system could reduce but not completely eliminate the influence of irrelevant information on subsequent information processing.

Altogether these findings support the existence of a temporal sequence involving different attentional processes that form the observed behavioral patterns during attentional capture: mechanisms that contribute to contingent attentional orienting on the one hand and to rapid reallocation of attention on the other hand.

The aim of the present study was to investigate the underlying attentional processes that cause the same-location effect (i.e., faster response for targets that appear on the same location as the preceding cue) in contingent attentional capture. One explanation would be that attention was initially drawn to the cued location and could not yet be withdrawn from that location by the time the target was presented (Theeuwes et al., 2000; Theeuwes, 2010). Another explanation would be that a spatial representation of the singleton cue was erroneously transferred into visual working memory. In line with the latter assumption, Remington et al. (2001) found that capture effects did not diminish with increasing stimulus-onset asynchronies (SOA; 100, 250, 450 ms) and outlasted the duration of the iconic memory store. They concluded that attention was shifted on-line to the cued location where it remained thereafter. Alternatively they proposed that despite any further allocation of attention, the processing of the color cue continues. However, it is difficult to draw these conclusions from behavioral results alone as they provide an indirect measurement of attentional allocation. Anyhow, these two explanations for the occurrences of the same-location effect should not be regarded as mutually exclusive.

**Figure 1:**
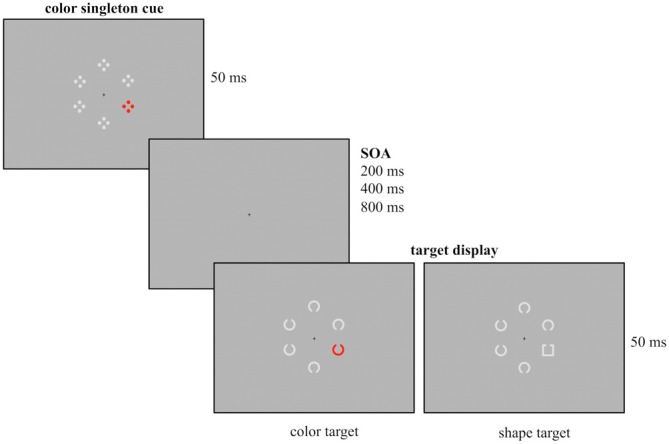
**Example of the experimental design used for the current study.** Participants had to report the gap position of the colored Landolt C (red, green, blue or yellow, here depicted in red) or the gray Landolt square that were presented among five gray Landolt Cs. Prior to target array onset a task-irrelevant cue array was presented for 50 ms followed by a randomly varying stimulus onset asynchrony (SOA; duration 200, 400 or 800 ms). The cue array was composed out of six stimuli with a colored singleton cue (red, green, blue or yellow, here depicted for the red condition) presented among five gray stimuli. Cue singletons and target singletons were randomly presented at the four lateralized positions. Thus, in 25% of the trials cue and target shared the same location.

To investigate the different assumptions concerning the same-location effect, we applied a spatial cuing paradigm similar to the one introduced by Eimer and Kiss (2008) with different SOA (200, 400, 800 ms) between cue and target array. We recorded ERPs evoked by the cue array in order to provide a continuous measurement of the allocation of attention. Cue displays containing a lateralized color singleton were presented prior to the target display (see Figure 1). Participants were instructed to ignore the task-irrelevant cue display, as the cue position did not allow for predicting the target position. They completed two different experimental blocks. In one condition, both cue and target singletons were defined by color (contingent condition). This should lead to capture of attention by the irrelevant cue. In the other condition, color singleton cue and target singleton did not share features so that the inhibition of the color singleton in the cue array should be facilitated (non-contingent condition).

Regarding contingent attentional orienting we predict faster response times and higher accuracy in cases where the cue and the following target occupy the same location. This pattern of results should only appear for the contingent color task of the experiment. In the shape task no such differences in response times and accuracy are expected. If spatial cuing effects in the contingent condition originate from a delay in the allocation of attention to the target we would expect them only for the shortest SOA condition (200 ms), when there is not enough time available to re-focus attention onto the central position after capture by the contingent color cue. If otherwise spatial information of the cue is maintained in working memory, faster responses on same location trials should last over the different SOA conditions and should also appear for the longer SOA conditions (Gibson and Amelio, 2000; Remington et al., 2001; Ansorge et al., 2013; Liao and Yeh, 2013).

On ERP level we expected the occurrence of an N2pc to the color cue in the contingent condition of the experiment, indexing the selection of information matching the current attentional control set. Regarding the non-contingent shape condition of the experiment we would predict an N2pc component in response to the color cue because both the cue and the target were singletons (i.e., a stimulus that has a unique characteristic presented with otherwise homogeneous stimuli) and participants could adopt a singleton search mode (see Bacon and Egeth, 1994; Lamy and Egeth, 2003). However, N2pc should be weaker in the shape condition compared to the color condition and its occurrence should be dissociated from the behavioral results (i.e., no faster response/ higher accuracy on same location trials in the shape task; Kiss et al., 2013).

If the same-location effect truly arises due to a lack of time to disengage attention from the irrelevant cue position we would expect that the inhibitory process indexed by the Pd component fails to be fully completed until target array presentation in the shortest SOA condition. For the longer SOAs where no capture effects are assumed, Pd should rapidly arise after N2pc to enable the suppression of the irrelevant information before the onset of the target array. In the study of Sawaki and Luck (2013), Pd to the cue occurred approximately 150 ms after target presentation. As cue and target were presented in close succession (300 ms) it could be assumed that suppression of the irrelevant information and perception of the relevant stimuli were forced to proceed in parallel. However it remains possible that Pd is directly linked to the onset of the target array. By adding different SOA conditions the present study will shed more light on the temporal progression of this inhibitory process: if the suppressive mechanism was directly linked to the occurrences of the target we would expect a Pd component at the time of search array presentation for all SOA conditions. Before Pd was labeled as an inhibitory process, Eimer and Kiss (2008) observed a positive deflection contralateral to a color cue replacing N2pc in the condition of their spatial cuing experiment in which no attentional capture was expected. The authors assumed that attention was voluntarily directed away from the distractor in situations where it does not match the attentional template. As we expect an N2pc component to the color cue, Pd should occur following N2pc rather than replacing it. This is also the pattern anticipated by Kiss et al. (2013). They proposed that attentional processing of irrelevant cues proceeds in two steps: initially, N2pc is triggered by each property that matches the current attentional template but attention is rapidly disengaged from the irrelevant information when it did not fully match the target defining features.

As mentioned above, another explanation for the same-location effect would be that spatial information of the cue is actively maintained in visual working memory. To our best knowledge, the contribution of working memory maintenance or transfer processes to the same-location effect in spatial cuing paradigms has not yet been investigated by means of ERPs. The storage of visuo-spatial information in working memory, when sensory information is no longer available, is reflected by a component labeled contralateral delay activity (CDA; Vogel and Machizawa, 2004; Ikkai et al., 2010). So far, this component was never considered in the context of spatial cuing paradigms. CDA is usually observed in the later time window of the retention interval prior to target array onset and is characterized as a sustained posterior negativity contralateral to the relevant information that has to be maintained. Therefore, provided that the same-location effect remains stable across the SOA conditions, we would expect a reliable CDA in the contingent condition. Due to its late onset this component could only be measured for the longest SOA condition (800 ms).

This investigation of the origins of the same-location effect in spatial cuing studies will contribute to a deeper understanding of attentional control processes in visual search.

## Materials and Methods

### Participants

Thirty-eight younger adults took part in the overall experiment. None of them reported any neurological or psychiatric problems. The data from three participants was excluded due to technical problems or excessive eye movements during the experimental session. Thus, 35 participants (17 females, *M_(age)_* = 24.34, *SD_(age)_* = 2.76, *Range* = 20–30 years) remained in the sample. All had normal or corrected to normal vision, were not color-blind (test by means of Ishihara color blindness test; Ishihara, 1991) and right-handed. Participants received course credits or a payment of 10€ per hour. The experiment was carried out in accordance to the Declarations of Helsinki. The local ethic committee of the Leibniz Research Centre for Working Environment and Human Factors endorsed the study and participants provided informed written consent.

### Stimuli and Procedure

Participants were seated in a dimly lit chamber in front of a 20” CRT monitor with a refreshing rate of 100 Hz. They viewed the stimuli from a distance of 145 cm. Presentation was controlled by VSG 2/5 graphic accelerator (Cambridge Research System, Rochester, UK). Following the cuing paradigm used by Eimer and Kiss (2008) at the beginning of each trial a cue array was presented for 50 ms and followed by a target array (50 ms presentation). Additionally, different SOA were implemented between cue and target array, randomly varying between 200, 400 or 800 ms. A small black fixation cross was continuously visible during presentation. The design of the cue array closely resembled the one of Experiment 1 from Eimer and Kiss (2008). The cue array was composed of six stimuli arranged in a circular array with each stimulus presented at a constant distance of 1.25° from fixation. It contained a color singleton cue (a red stimulus among gray stimuli) that was randomly presented at lateral positions and never at central positions above or below the fixation cross. Each of the six cue stimuli consisted of four outlined dots that were arranged in form of a diamond.

The following circular target array included six stimuli out of which one was the singleton target. In the color condition, the target array was composed out of six Landolt Cs (0.625° diameter, 0.125° thick, 0.125° gap) with one stimulus presented in red and the remaining stimuli presented in gray. In the shape condition the target array consisted of five gray Landolt Cs and a gray Landolt square (0.55° × 0.55°, 0.125° thick, 0.125° gap). All stimuli had a gap at their bottom or top. Like the colored cue stimuli, the target stimuli were randomly presented at one of the four lateral positions. Participants had to report the position of the gap by button press with the index finger of the left or the right hand (e.g., gap at the top right button press, gap at the bottom left button press), with the assignment of gap position and response hand counterbalanced across participants. The responses were measured with force keys and were recorded along with the EEG.

Participants performed two consecutive experimental blocks (color condition, shape condition), with each block including 960 trials. After every 160 trials participants had a 2-min break, so that each condition consisted of six subblocks. The block order (color vs. shape condition) was counterbalanced across participants. All stimuli were presented with equal probability at one of the four lateral positions, so that the color singleton cues were spatially uninformative with respect to target location (25% validity). The red and gray stimuli were presented on a dark gray background (10 cd/m^2^) and were matched for luminance (25 cd/m^2^). Luminance was calibrated with ColorCAL MKII Colorimeter (Cambridge Research Systems). Therefore the luminance values were measured on the monitor and then adjusted in the VSG graphic accelerator.

While for 19 out of the 35 participants the cue and target singletons (in the contingent condition) were always red stimuli (CIE color coordinates = 0.566/0.376) among gray stimuli (CIE color coordinates = 0.287/0.312), the singleton color was red, green (CIE color coordinates = 0.292/0.574), blue (CIE color coordinates = 0.168/0.131) or yellow (CIE color coordinates = 0.384/0.477; randomized) for the remaining 16 participants. The new colors were also matched for luminance (25 cd/m^2^) and each color was presented equally probable. The color of the cue and the following target were the same for each trial. We conducted this second experiment to investigate if contingent orienting is affected when the attentional control set for a red target is extended to a broader control set for several color singleton targets. Even if participants now had to respond to different colors, the behavioral and ERP results did not differ between the two experimental groups. This is in line with earlier findings in cuing experiments showing that contingent capture is also triggered when the attentional template is less specific and set for more than one color (Folk et al., 1992; Folk and Anderson, 2010; Harris et al., 2015). Thus, we decided to combine the data of the two samples to have more statistical power for the analyses.

### Data Analysis

#### Behavioral Data

Errors in the current experiment involved missed responses (no response within 1000 ms following the target array) and incorrect assignments of response side to the orientation of the Landolt singletons. Responses with response times (RTs) shorter than 150 ms were categorized as fast guesses and were also treated as errors. Separate analyses of variance (ANOVA) with the within-subjects factors *condition* (color, shape), *cue-target location* (same quadrant, vertical quadrant, horizontal quadrant, diagonal quadrant) and *SOA* (200 ms, 400 ms, 800 ms) were used to test for differences on mean RTs and error rates.

#### EEG Data

The EEG was recorded using 60 active Ag/AgCl electrodes (ActiCap; Brain Products, Gilching, Germany) according to the extended 10/20 System (Pivik et al., 1993). To assess the vertical and horizontal EOG (electrooculogram), two electrode pairs were affixed at the outer canthi of each eye and above and below the left eye. EEG and EOG were sampled at 1000 Hz by a BrainAmp DC-amplifier with a low-pass filter of 250 Hz. Online reference site was electrode Fpz. Impedances were kept below 10 kΩ. All analyses were conducted using MATLAB^®^ and the related packages EEGLAB (Delorme and Makeig, 2004) and ERPLAB (Lopez-Calderon and Luck, 2014) for EEG/ERP data analysis. The data were re-referenced offline to the average of the left (TP9) and right (TP10) mastoid. Signals were filtered offline with a 0.5 Hz high-pass and a 30 Hz low-pass filter. Epochs beginning 500 ms before and ending 2200 ms after cue display onset were chosen to obtain the averaged ERP waveforms. The 200 ms pre-stimulus period served as the baseline. Only trials that contained a correct response were chosen for further analyses. Trials with RTs shorter than 150 ms (fast guesses) or longer than 1000 ms were also excluded. To correct for eye-movement artifacts and discontinuities in the EEG data an independent component analysis (ICA) was run. Every fourth trial was used as the basis of data for ICA. ADJUST (Mognon et al., 2010) was then used to automatically exclude artifacts in the ICA data. Participants with excessive eye movements were excluded from the analysis, as this behavior constitutes a violation of the task instructions and would therefore distort the results of the current study. To detect eye movements we measured saccades at the left and right hEOG channels separately for each task and SOA condition in the respective time window between cue and target array onset. All trials in which hEOG activity exceeded a threshold of 25 μV or −25 μV were marked as containing eye movement artifacts. Participants were excluded from all further analysis when they showed saccades in more than 10% of all trials.

To measure the N2pc, Pd and CDA components, we computed the activation contra- and ipsilateral to the singleton cue. Event related lateralizations (ERLs) were measured by subtracting the ipsilateral from the contralateral signal. Thus ERLs were calculated the same way the lateralized readiness potential is computed (Coles et al., 1988; Wascher and Wauschkuhn, 1996). The components were measured at electrodes PO7 and PO8. Following the approach of Sawaki et al. (2012) rather than computing mean amplitudes, we measured the positive or negative area under the ERL difference wave over the time interval of interest for each component and averaged it across participants. From calculating these area measurements we will always receive values greater than zero that could also result from random noise in the data. As a reference for the statistical significance of the measured area values we calculated a distribution of area values that would be expected if there were only random lateralized activation in the data. To estimate this distribution we conducted a permutation test: we randomly assigned the side of the target for each trial, computed the resulting negative or positive area value under the ERL for each participant and then averaged across participants. This procedure was repeated 1000 times with different randomizations. If the measured area value in the original data was higher than 95% of values from this random distribution, we assumed a significant difference from chance level. The negative area in the time window from 170–290 ms was measured to assess N2pc. ANOVAs for the within-subjects factors *condition* (color, shape) and *SOA* (200, 400, 800 ms) were conducted to test for differences in N2pc area values. The SOA variation revealed that there were two independent Pd effects, a first effect time-locked to cue display onset (i.e., Pd-early) and a later one appearing after the onset of the target display (i.e., Pd-late). Pd-early was analyzed as the positive area under the difference wave in the time interval from 300–400 ms for all three SOA conditions. For Pd-late only the 400 ms SOA condition was considered for the statistical analysis, because Pd-late overlapped with Pd-early in the 200 ms SOA condition and with the later CDA in the 800 ms SOA condition. A time window from 500–600 ms was used for Pd-late analyses. The negative area related to CDA was measured between 500–800 ms after cue array onset. Area measurements of the CDA were only conducted for the 800 ms SOA conditions. In the remaining SOA conditions (200 ms and 400 ms), the occurrence of the CDA coincided with the target-related perceptual processing. Partial eta squared ($\eta_{p}^{2}$) is reported as an indication of effect size. *P*-values of <0.05 were considered statistically significant.

To exclude the possibility that the late Pd process simply reflects lateralized sensory processing elicited by the target array, we also examined Pd-late dependent on the cue-target location. A contralateral positive enhancement of P1 with this positivity lasting throughout the N1 latency range was shown to reflect attentional gain control mechanism to facilitate sensory processing for stimuli at attended locations (Heinze and Mangun, 1995). Therefore, we measured the contralateral and ipsilateral P1/N1 peak amplitude in response to the target array. We conducted the analyses only for the 400 ms SOA condition because for the remaining conditions the Pd overlapped with other attentional processes (see above). The P1 peak was analyzed in the time window between 480–550 ms and N1 peak between 550–640 ms after cue array onset. As we could not observe any difference between ERPs of same and vertical quadrant trials we combined these trials to an overall same side condition. The same applied for horizontal and diagonal quadrant trials so that they were combined to different side trials. This ensured an appropriate amount of trials in each condition and more power for statistical analysis. Statistical significance was assessed by means of an ANOVA with the factors *condition* (color, shape), *asymmetry* (contralateral, ipsilateral referred to the cue) and *target position* (same side, different side referred to the cue).

## Results

### Behavioral Results

**Figure 2:**
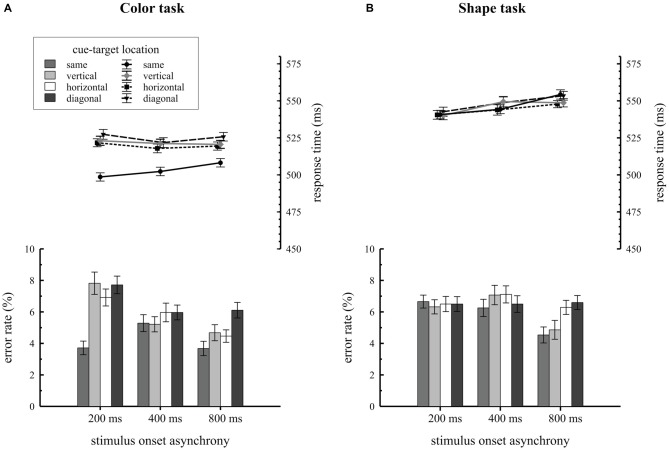
**Behavioral results depending on stimulus-onset asynchronies (SOA) conditions and cue target location.** Response times and error rates are separately shown for the color **(A)** and the shape **(B)** task.

Mean RTs and error rates for trials, where the target was presented at the same, vertical, horizontal or diagonal quadrant relative to the cue, are separately shown for the two task types in Figure 2. Participants revealed overall faster RTs in the color task (*M* = 517 ms; *SE* = 1.5) compared to the shape task (*M* = 546 ms; *SE* = 1.5), *F*_(1,34)_ = 21.2, $\eta_{p}^{2}$ = 0.38, *p* < 0.001. As expected, the significant two way interaction of condition by cue-target location, *F*_(3,102)_ = 28.2, $\eta_{p}^{2}$ = 0.15, *p* < 0.001, proved that attentional capture effects on behavior were only found for the color task but not for the shape task. In the color task, participants responded faster on same quadrant trials (*M* = 503 ms; *SE* = 1.8) compared to vertical (*M* = 522 ms; *SE* = 1.8), horizontal (*M* = 520 ms; *SE* = 1.8) and diagonal (*M* = 525 ms; *SE* = 2.0) quadrant trials, *F*_(3,102)_ = 44.6, $\eta_{p}^{2}$ = 0.19, *p* < 0.001. Overall RTs were about 19 ms faster when cue and target shared the same location. This pattern of results was not found for the shape task: here RTs did not differ with respect to cue-target location (same quadrant: *M* = 546 ms; *SE* = 2.0; vertical quadrant: *M* = 546 ms; *SE* = 2.0; horizontal quadrant: *M* = 544 ms; *SE* = 1.9, diagonal quadrant: *M* = 548 ms; *SE* = 2.1), *F*_(3,102)_ = 1.3, $\eta_{p}^{2}$ = 0.01, *p* = 0.28. As the three way interaction of condition by cue-target location by SOA did not reach significance, *F*_(6,204)_ = 1.3, $\eta_{p}^{2}$ = 0.006, *p* = 0.28, there was no variation in spatial cuing effects depending on the time interval between cue array and target array onset.

Error rate analysis revealed a significant condition by cue-target location by SOA interaction, *F*_(6,204)_ = 4.4, $\eta_{p}^{2}$ = 0.02, *p* < 0.001. Additional ANOVAs were conducted for each SOA condition to break down this effect. For the 200 ms SOA condition we observed a significant task by cue-target location interaction, *F*_(3,102)_ = 10.7, $\eta_{p}^{2}$ = 0.08, *p* < 0.001. In this SOA condition participants made fewer errors on same quadrant trials relative to all different location trials (vertical, horizontal, diagonal quadrants) but the effect only showed up for the color task. No comparable results were found for the 400 ms SOA condition, *F*_(3,102)_ = 0.7, $\eta_{p}^{2}$ = 0.007, *p* = 0.58 and the 800 ms SOA condition, *F*_(3,102)_ = 1.9, $\eta_{p}^{2}$ = 0.02, *p* = 0.13.

### ERP Results

**Figure 3:**
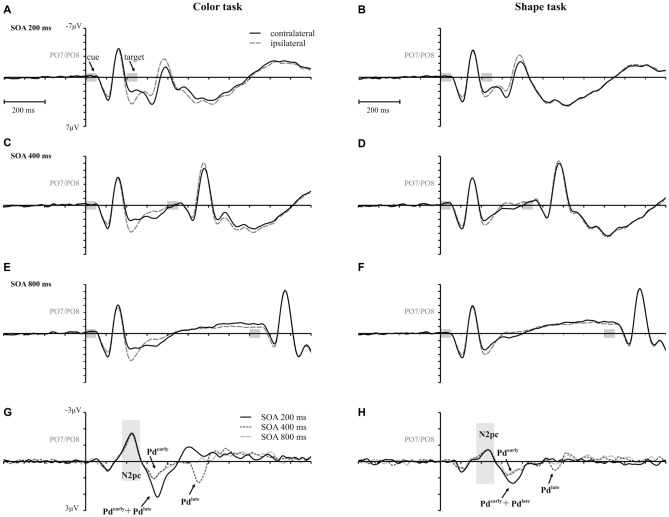
**Event-related potentials (ERPs), time-locked to cue array onset at posterior electrodes (PO7/PO8) for each SOA condition are separately shown for the color and the shape task**. Contralateral and ipsilateral waveforms are depicted relatively to the side of the color singleton cue **(A–F)**. The lower row shows the difference waves (event related lateralizations, ERLs) for the color **(G)** and the shape **(H)** task. Time points of the cue and the target array are highlighted in gray.

**Figure 4:**
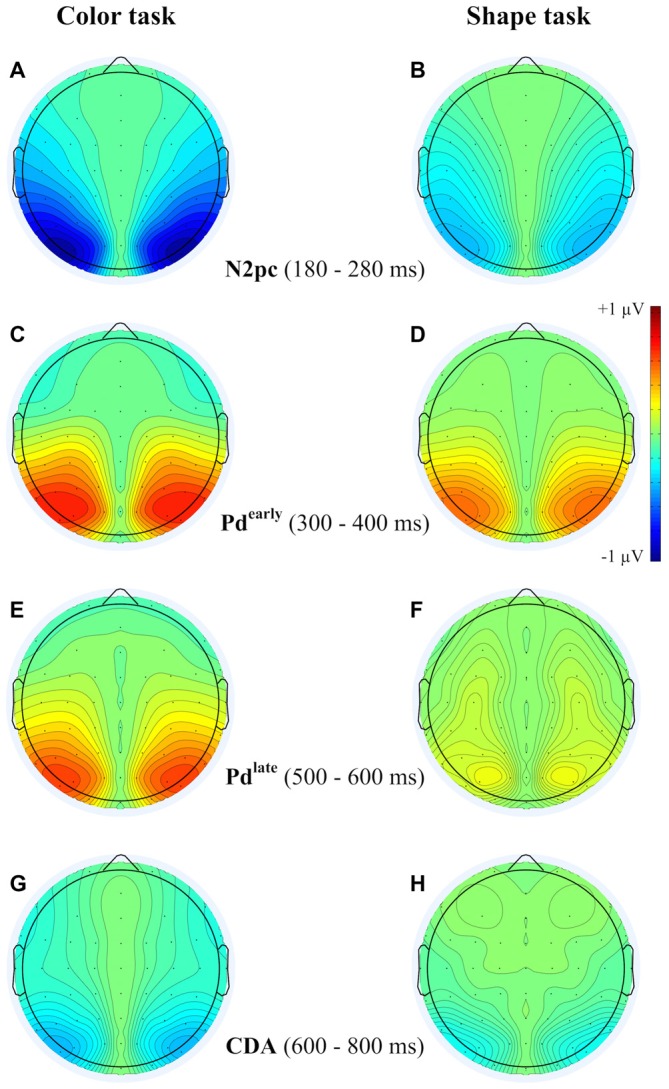
**Topographies from the ERLs during the time interval of N2pc (180–280 ms) **(A,B)**, Pd-early (300–400 ms) **(C,D)**, Pd-late (500–600 ms) **(E,F)** and CDA (600–800 ms) **(G,H)** separately depicted for the color and the shape task**.

**Figure 5:**
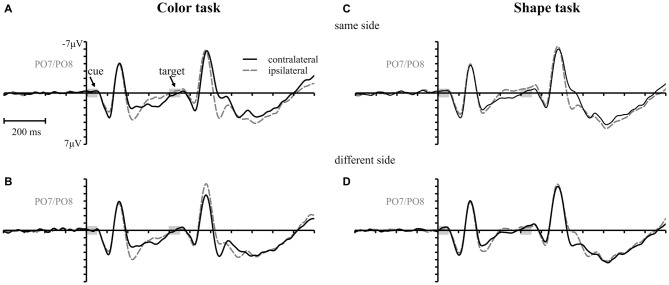
**Target elicited P1/N1 components.** Waveforms contra- and ipsilateral to color cues presented at the same (same and vertical quadrant) **(A,C)** and different side (horizontal and diagonal quadrant) **(B,D)** with respect to the color and shape target for the 400 ms SOA.

Figure 3 shows the waveforms contra- and ipsilateral relative to the location of the color cue and the ERLs for the two task types and the three SOA conditions. Critical values and observed values of the permutation analyses for N2pc, Pd and CDA components are shown in Table 1. Additionally, scalp map topographies are depicted for both task types and the corresponding time windows in Figure 4. Activation contra- and ipsilateral to cues presented at same sides (same and vertical quadrant) and different sides (horizontal and diagonal quadrant) referred to the color and shape target are depicted in Figure 5 to illustrate the target-elicited P1/N1 components in the 400 ms SOA.

**Table 1:** **Critical and observed values of the area permutation analyses for N2 posterior contralateral (N2pc), distractor positivity (Pd) and contralateral delay activity (CDA) components for the respective SOA conditions**.

	Color task		Shape task	
Component	Critical	Observed	Critical	Observed
**N2pc**
SOA 1	0.025	0.113*	0.025	0.040*
SOA 2	0.026	0.104*	0.027	0.045*
SOA 3	0.026	0.099*	0.026	0.050*
**Pd early**
SOA 2	0.022	0.070*	0.023	0.061*
SOA 3	0.022	0.076*	0.022	0.055*
**Pd late**
SOA 2	0.023	0.071*	0.024	0.019
**CDA**
SOA 3	0.062	0.090*	0.066	0.056

*When the observed value is greater than the critical value, 95% of the values from the permutation distribution are exceeded and significance of the component is assumed*.

A reliable N2pc component, indicative of attentional capture by the color singleton cue was observable in all conditions. It starts at about 170 ms and showed up as a contralateral negativity over parieto-occipital electrode sites (Figures 4A,B). For the color task this activation was significantly more pronounced than in the shape task, *F*_(1,34)_ = 23.0, $\eta_{p}^{2}$ = 0.40, *p* < 0.001. As was to be expected, there were no differences in N2pc between the three SOAs, *F*_(2,68)_ = 0.3, $\eta_{p}^{2}$ = 0.004, *p* = 0.78 just as no significant condition by SOA interaction, *F*_(2,68)_ = 2.7, $\eta_{p}^{2}$ = 0.01, *p* = 0.073. Permutation tests indicated that activation in the time window of the N2pc (170–290 ms) was significantly different from zero for the color and the shape task (see Table 1).

Pd-early could be observed as a positive deflection contralateral to the singleton cue following N2pc, beginning approximately 300 ms after cue array onset. Topographies show that this effect was limited to lateral parieto-occipital scalp sites (Figures 4C,D). What becomes apparent from the ERLs is that there was a stronger Pd effect for the shortest SOA relative to both longer SOA conditions (Figures 3G,H). This effect was due to an additional inhibitory process during target array onset that could also be observed in the 400 ms SOA condition as a positive deflection in the difference wave simultaneously to the perceptual processing of the target array. When all three SOA conditions were included, analysis revealed a significant condition by SOA interaction, *F*_(2,68)_ = 8.1, $\eta_{p}^{2}$ = 0.10, *p* < 0.001 due to this additional positivity in the first SOA condition. Thus, further statistical analysis of Pd-early was only conducted for the 400 ms and 800 ms SOA condition, where its effects could be considered more genuinely. There were no main effects of condition, *F*_(1,34)_ = 2.6, $\eta_{p}^{2}$ = 0.07, *p* = 0.11, or SOA, *F*_(1,34)_ = 0.003, $\eta_{p}^{2}$ = 0.0001, *p* = 0.96, as well as no significant condition by SOA interaction, *F*_(1,34)_ = 3.5, $\eta_{p}^{2}$ = 0.09, *p* = 0.07. This suggests that the Pd-early effect was of similar size regardless of whether there was a contingency in attentional control sets or not. Area analysis revealed a significant effect for the two task and SOA conditions (time window: 300–400 ms; see Table 1).

As mentioned above, there was a second contralateral positivity (Pd-late) that appeared after target presentation and overlapped with Pd-early in the 200 ms SOA condition. Comparable to Pd-early, Pd-late was limited to lateral parieto-occipital scalp sites (Figures 4E,F). It was significantly more pronounced in the color task than in the shape task, *F*_(1,34)_ = 17.1, $\eta_{p}^{2}$ = 0.33 *p* < 0.001. Permutation analysis supported a significant effect in the color but not in the shape task (time window: 500–600 ms; Table 1). To ensure that this late Pd effect was not confounded with target-related sensory processes we analyzed P1/N1 components to the target dependent on cue location (same side, different side). For P1, ANOVA results revealed a significant condition by asymmetry by target position interaction, *F*_(1,34)_ = 4.6, $\eta_{p}^{2}$ = 0.12, *p* < 0.05. When the color and the shape task were considered separately, we found a significant two-way interaction of asymmetry by target position for the color task, *F*_(1,34)_ = 13.3, $\eta_{p}^{2}$ = 0.28, *p* < 0.001 but not for the shape task, *F*_(1,34)_ = 2.3, $\eta_{p}^{2}$ = 0.06, *p* = 0.14. In the color task the P1 was contralaterally enhanced for same side trials (see Figures 5A,B). N1 peak analysis revealed also a significant three-way interaction of condition by asymmetry by target position, *F*_(1,34)_ = 8.2, $\eta_{p}^{2}$ = 0.19, *p* < 0.01. For the color task this effect remained significant, manifesting in the significant asymmetry by target location interaction, *F*_(1,34)_ = 23.0, $\eta_{p}^{2}$ = 0.40, *p* < 0.001. From Figures 5A,B, it can be seen that for the color task the N1 was contralaterally more positive on different side trials compared to same side trials. This was not the case for the shape task, *F*_(1,34)_ = 0.4, $\eta_{p}^{2}$ = 0.01, *p* = 0.55.

As evident in Figure 3E, for the 800 ms SOA preceding target array onset, we observed a sustained negative deflection contralaterally to the cued location that was obviously pronounced for the contingent color task. This increased negativity was observable at parietal and occipital electrodes and slightly spread out over temporal scalp sites (Figures 4G,H). CDA in the color task differed significantly from CDA in the shape task, *F*_(1,34)_ = 4.4, $\eta_{p}^{2}$ = 0.11, *p* < 0.05. Permutation test (time window: 500–800 ms) verified a significant CDA effect for the color but not for the shape task (Table 1). Consequently, in the color task, a reliable CDA was triggered through the color singleton cue whereas the shape task did not lead to a sustained contralateral activation prior to target array onset.

## Discussion

How does information that is spatially and temporally irrelevant influence the processing of subsequently presented information? The present study investigated the different processing steps contributing to contingent attentional orienting and rapid reallocation of attention in spatial cuing paradigms by means of event related potentials. An irrelevant color cue, which was either contingent or non-contingent on attentional sets, was presented prior to a target array with different SOAs (200, 400, 800 ms).

Behavioral results were consistent with the assumption that capture is contingent on attentional sets: when cue and target were composed of the same defining properties, RTs were faster when they shared the same location (i.e., same-location effect) compared to conditions with targets that were presented at different locations referred to the cue. In cases where the cue did not match the top-down search goal, RTs did not differ regardless of whether the color cue appeared in the target location or elsewhere. This suggests that the irrelevant color cue did not attract attention. Alternatively, disengagement from the location of the irrelevant cue could have been proceeded relatively fast in this condition (Theeuwes et al., 2000; Kiss et al., 2013).

The time course of attentional capture was investigated by using different time intervals between cue and target presentation. Thereby we wanted to determine the origins of the same-locations effect: insufficient time to withdraw attention from the irrelevant cue position or maintenance of spatial information in working memory. RTs revealed no evidence for a modification of the position effect over time, but participants made fewer errors on same location trials compared to different location trials only in the shortest SOA condition of the color task. In such a case, time to disengage from the cued location was very restricted so that attention should still have been focused on that location by the time the target appeared. Thus, the amount of time available after the presentation of irrelevant objects has an impact on the precision of visual search in a subsequent target display, but only when cue and target singletons were composed of the same defining properties. However, the RT findings indicate that the same-location effect cannot be solely based on insufficient time available for the re-allocation of attention after capture. Like in previous studies we found persisting capture effect in RTs (Gibson and Amelio, 2000; Remington and Folk, 2001; Ansorge et al., 2013; Liao and Yeh, 2013). This might point towards the formation of a sustained spatial representation of the color cue.

A look at the ERPs underpins the findings in the behavioral data and helps to clarify what happens during attentional processing of the irrelevant cue. As expected, N2pc component was found in response to the color cue for all SOA conditions and was markedly pronounced when the cue was contingent on attentional control sets. We also found an N2pc component to the color cue in the non-contingent shape task, albeit smaller than in the color task. This corresponds to the assumption that participants used singleton search mode to accomplish the task. However, the presence of N2pc in this condition was not linked to attentional capture effects in the behavioral data. Even though this result contrasts with the findings of some previous studies (Eimer and Kiss, 2008; Leblanc et al., 2008), there are also studies reporting similar findings (e.g., Kiss et al., 2013). Kiss et al. (2013) hypothesized that this dissociation between ERP and behavioral findings in the non-contingent task stems from rapid disengagement applied to stimuli that did not fully match the sought-after object. The results imply that capture does not occur following the all-or-nothing principle but that visual input might have to exceed a certain threshold to trigger attentional capture on the behavioral level. An explanation for the reduced N2pc amplitude in the shape task might be that attentional capture only appeared on a subset of trials leading to a reduction in amplitude when averaging across all trials. Otherwise, it could be assumed that N2pc amplitudes are generally pronounced in the color compared to the shape task. Töllner et al. (2010) could show that N2pc amplitudes are enhanced for targets matching the dimension of the preceding cue. Participants intentionally weighted the dimension of the cue leading to a distribution of processing resources in favor of this dimension. This intentional preparation facilitated the processing of the target when it was presented in the same dimension as the preceding cue. In the current experiment, participants had to prepare for upcoming targets defined in the dimension “color” or “shape”. Following the assumption of Töllner et al. (2010), the color cue “benefited” from this intentional weight distribution in the contingent condition resulting in enhanced N2pc amplitudes in response to the cue.

A first Pd time-locked to cue-display onset arose in the time window after N2pc and was found in all conditions of the experiment (i.e., Pd-early). Unlike N2pc, this contralateral positivity did not vary with the attentional set. In the non-contingent condition, this process seemed to be sufficient to prevent attentional capture at the behavioral level. But, as mirrored by the persisting same-location effect in the contingent condition, Pd-early was not able to neutralize the impact of cue singleton location on target processing. Thus, rather than reflecting a mechanism that prevents the passage of the visuo-spatial cue information into working memory, this Pd could be interpreted as an inhibitory process to neutralize attentional orienting to the irrelevant cue. This suppression should allow for the rapid disengagement of the attentional focus from the cued location and thus enabling the re-direction to fixation to guarantee the appropriate processing of the upcoming target. For the first SOA, this Pd-early overlapped with a later contralateral positivity effect after target display onset. These effects added up, leading to the impression of a stronger overall Pd in the first compared to the second and third SOA conditions (see Figures 3G,H). As the target appeared quickly after the cue array in this condition, the two processes were forced to proceed in parallel. This might have caused an insufficient completion of these mechanisms explaining the stronger capture effects in 200 ms SOA condition based on error rates. But for the second SOA condition, where there was more time available, we could observe both processes independently of one another: Pd-early arose in the 300–400 ms time window after cue array onset and was followed by a later effect in the 500–600 ms time window (i.e., Pd-late; note the 200 ms temporal offset between SOA condition 1 and 2) by the time the target array was already presented (see Figures 3G,H). For the third SOA condition Pd-late was covered by CDA that lasted until target array onset, so that no clear change in polarity was observable. However, in the 900–1000 ms time window negativity returned to baseline pointing towards the occurrence of an overlapping positivity (see Figure 3G). This implies a regularity of this late positivity as it occurs approximately 150 ms after the presentation of the target display in each SOA condition. Whereas Pd-early remained stable throughout all conditions, Pd-late was modified depending on the attentional set with a stronger effect in the contingent condition of the experiment. Thus, Pd-late should be associated with the active inhibition of the spatial cue information. As expected this suppressive mechanism seems to be directly linked to the occurrence of the target in order to compensate for interference with the irrelevant information that was still represented by the time the target display appeared. The fact that Pd-early and Pd-late revealed an additive effect in the 200 ms SOA condition of the current experiment might point towards the notion that their underlying mechanisms are physiologically distinct. Yet, this does not exclude that both mechanisms interact concerning their impact on visual search performance.

However, it remains possible that this late positivity reflects sensory gain control rather than an inhibitory process as it arose together with the perception of the target array. In such a case we would expect a contralateral enhancement of P1 for stimuli at the attended side that persists throughout the N1 time range (Heinze and Mangun, 1995). Regarding P1 the current findings were in line with the assumptions of Heinze and Mangun as we observed a contralaterally enhanced P1 amplitude for color targets that were presented at the same location as the preceding cue. Larger P1 amplitudes at contralateral sides referred to an attended object were also reported in a study of Gramann et al. (2010). They revealed an enhancement of P1 amplitude when cue and target presented at the same location shared dimensions compared to conditions when there was no match in dimension. Even though P1 findings of the current investigation fit well with the attentional gain control account, there is still ambiguity regarding the modulation of N1 in the current study. In fact, N1 was only affected for color targets presented at different sides referred to the cue (Figure 5B). In this condition we could observe a contralateral reduction of N1. No such significant asymmetries for P1/N1 were found in the shape task (Figures 5C,D). On the one hand it is possible that the P1 modulation for same-location targets in the color task really reflected gain control and that inhibitory mechanisms in the N1 time window were only engaged when the target was presented at a different location than the preceding cue. This would provide an explanation for the contralaterally reduced N1 for different location trials in the color task. On the other hand we could assume that the perception of the target array triggered the inhibition of the cue-representation and that this inhibitory process shifted in latency when the color target was presented at an unattended location.

Additionally, the Pd-late results strongly correspond to the Pd findings of Sawaki and Luck (2013) who also observed a contralateral positivity at about 150 ms after the onset of the target array. Furthermore, their ERP analysis revealed exactly the same pattern of the early sensory P1/N1 components as the current study. Sawaki and Luck interpreted this positivity as the termination of attentional priority at a formerly attended location to enable the reorienting towards the target. The authors assumed that this effect reflects an inhibitory response to the color cue and not to the target because it occurred 90 ms after the onset of the target. Furthermore, there exists no exact time frame regarding the Pd effect providing the possibility that this mechanism started relatively late and coincided with the perception of the target in this specific experimental design. By implementing different SOA conditions the current experiment however could show that the late suppressive mechanism was directly linked to the onset of the target. Pd-late mirrored the active suppression of the color cue’s location but is triggered through the occurrences of relevant information. The fact that Sawaki and Luck could not observe a Pd-early might be due to differences in the temporal task demands of the experiments. In the current study, the time interval between cue and target array varied randomly and participants could not exactly anticipate the occurrence of the relevant stimulus. Therefore, they had to rapidly suppress the selected cue information (indexed by Pd-early) to be prepared for the upcoming target display that could appear within 200 ms after cue display onset. The study of Sawaki and Luck (2013) was based on a constant cue-target interval of 300 ms. The rapid suppression of attentional capture might not be required to the same extent with such temporal task demands. Furthermore, the cue display in the current study contained a color singleton that should more likely have triggered a shift of spatial attention to its location than the four-color cue display used by Sawaki and Luck (2013).

Interestingly, we could observe a sustained activation contralateral to the color cue, namely a CDA component, only in the contingent condition of the task. So far, the current study has been the first to investigate the relevance of this component in the context of contingent attentional orienting. The occurrence of the CDA component confirms that in cases where attentional capture was contingent on attentional control sets, spatial information of the irrelevant object was encoded in visuo-spatial working memory. The maintenance of the spatial information explains why there was a same-location effect not only in the 200 ms SOA condition but also for longer SOAs. The sequence of processes observed in the current study is similar to those reported by other studies (Mazza et al., 2007; Jolicoeur et al., 2008; Töllner et al., 2013): an N2pc component [also referred to as posterior contralateral negativity (PCN)] that reflects the selection of object information is followed by a CDA component, indexing active maintenance of the selected information. But CDA arises only in cases when there is a match between the current attentional template and the selected information. In accordance with the assumptions of Kiss et al. (2013), this points towards two filtering stages regarding the passage of information into working memory: an early selection process represented by N2pc and a later, more conservative filtering stage determining working memory access (here associated with CDA). Finding a CDA component in response to the color cue provides evidence that the same-location effect is based on working memory representations that survive the first inhibition of the cued location (Pd-early) and are actively maintained until the truly relevant information occurs. Only then, these representations are suppressed (Pd-late) to enable the rapid perception of the target.

It should be mentioned that we observed an asymmetry of the P1 component in response to the cue array. As all stimuli were matched for luminance this asymmetry could hardly be explained through a sensory unbalanced presentation of the stimuli in the cue array. However, P1 asymmetries did not vary depending on the different experimental conditions and did not influence the pattern of attentional capture: in the shape task, we neither found a CDA component nor did we observe a same-location effect in the behavioral data. Thus, this early effect cannot account for the behavioral results and task-specific ERP findings in the current study.

In conclusion, the results of the current investigation provided new insights about the attentional processes triggered by irrelevant information. It has long been known that even though information is currently irrelevant, it can draw attention when it matches the attentional control sets. It seems that there exists a certain gradation of attentional capture as it could occur at the electrophysiological level but does not necessarily have to affect behavior. Furthermore, the current study provides new evidence that the actually irrelevant information is automatically transferred into visuo-spatial working memory. In such a way this information remains able to influence further information processing which may explain the occurrence of the same-location effect in spatial cuing tasks even for long SOAs. Nevertheless, the attentional system can overcome attentional capture relatively fast, allowing the re-orienting to the relevant information and the flexible adaptation of behavior. The findings of the present study point towards the existence of two independent processes that enable rapid recovery from capture: a first process that reflects the inhibition of the selected cue information to enable the re-orienting of the attentional focus (i.e., Pd-early) and a second one that is engaged to actively inhibit the irrelevant information during target resolution (i.e., Pd-late). As this later process does not occur until the target is present, it might be only engaged to facilitate target processing but not to neutralize the former allocation of attention. However, Pd seems to be an important correlate of cognitive control mechanisms that is crucial to bring attention “back on track”.

For a better understanding of the attentional processes reflected by the Pd component it would be helpful to create experimental designs in which cognitive control must be adapted to improve performance. Recently Sawaki et al. (2015) investigated how attentional mechanisms responded in situations where rewards were expected and found a Pd to high incentive cues. Thus, these high reward-promising cues were actively suppressed to minimize interference with the upcoming target, thereby enabling prioritization of the target and optimal performance. This suggests that cognitive control processes may be flexibly adapted depending on the demands and priority of the current situation. That the process of attentional capture is altered in people with an impaired flexibility regarding cognitive control was already shown by studies including samples of elderly people (Lorenzo-Lopez et al., 2008; Wiegand et al., 2014) or people with psychiatric diseases (Luck et al., 2006; Cross-Villasana et al., 2015). However, only few studies based on inter-individual differences in cognitive control mechanisms investigated the concept of contingent attentional orienting and measured the respective electrophysiological correlates (Lien et al., 2011; Verleger et al., 2014). Additional research in this matter might be worthwhile in order to learn more about the nature of processes underlying contingent attentional orienting.

## Author Contributions

DS and CM conceived the experimental design and analyzed the behavioral and electrophysiological data. CM carried out the experiment, collected the data and wrote the manuscript. Interpretation, revision and final editing of the work were performed by DS, EW and CM.

## Conflict of Interest Statement

The authors declare that the research was conducted in the absence of any commercial or financial relationships that could be construed as a potential conflict of interest.

## References

[B1] Ansorge U. Priess H. W. Kerzel D. (2013). Effects of relevant and irrelevant color singletons on inhibition of return and attentional capture. Atten. Percept. Psychophys. 75, 1687–1702. 10.3758/s13414-013-0521-2 24027028

[B2] Bacon W. F. Egeth H. E. (1994). Overriding stimulus-driven attentional capture. Percept. Psychophys. 55, 485–496. 10.3758/bf03205306 8008550

[B3] Baddeley A. (1996). The fractionation of working memory. Proc. Natl. Acad. Sci. U S A 93, 13468–13472. 8942958 10.1073/pnas.93.24.13468 PMC33632

[B4] Baddeley A. (2010). Working memory. Curr. Biol. 20, R136–R140. 10.4324/9781315793252 20178752

[B5] Coles M. G. Gratton G. Donchin E. (1988). Detecting early communication: using measures of movement-related potentials to illuminate human information processing. Biol. Psychol. 26, 69–89. 10.1016/0301-0511(88)90014-2 3061481

[B6] Cross-Villasana F. Finke K. Hennig-Fast K. Kilian B. Wiegand I. Muller H. J.. (2015). The speed of visual attention and motor-response decisions in adult Attention-Deficit/Hyperactivity Disorder. Biol. Psychiatry 78, 107–115. 10.1016/j.biopsych.2015.01.016 25773661

[B7] Delorme A. Makeig S. (2004). EEGLAB: an open source toolbox for analysis of single-trial EEG dynamics including independent component analysis. J. Neurosci. Methods 134, 9–21. 10.1016/j.jneumeth.2003.10.009 15102499

[B8] Desimone R. Duncan J. (1995). Neural mechanisms of selective visual attention. Annu. Rev. Neurosci. 18, 193–222. 10.1146/annurev.neuro.18.1.193 7605061

[B9] Eimer M. Kiss M. (2008). Involuntary attentional capture is determined by task set: evidence from event-related brain potentials. J. Cogn. Neurosci. 20, 1423–1433. 10.1162/jocn.2008.20099 18303979 PMC2564114

[B10] Folk C. L. Anderson B. A. (2010). Target-uncertainty effects in attentional capture: Color-singleton set or multiple attentional control settings? Psychon. Bull. Rev. 17, 421–426. 10.3758/pbr.17.3.421 20551369

[B11] Folk C. L. Remington R. W. Johnston J. C. (1992). Involuntary covert orienting is contingent on attentional control settings. J. Exp. Psychol. Hum. Percept. Perform. 18, 1030–1044. 10.1037/0096-1523.18.4.1030 1431742

[B12] Folk C. L. Remington R. W. Wright J. H. (1994). The structure of attentional control: contingent attentional capture by apparent motion, abrupt onset and color. J. Exp. Psychol. Hum. Percept. Perform. 20, 317–329. 10.1037/0096-1523.20.2.317 8189195

[B13] Gramann K. Töllner T. Müller H. J. (2010). Dimension-based attention modulates early visual processing. Psychophysiology 47, 968–978. 10.1111/j.1469-8986.2010.00998.x 20233340

[B14] Gibson B. S. Amelio J. (2000). Inhibition of return and attentional control settings. Percept. Psychophys. 62, 496–504. 10.3758/bf03212101 10909240

[B15] Harris A. M. Becker S. I. Remington R. W. (2015). Capture by colour: evidence for dimension-specific singleton capture. Atten. Percept. Psychophys. 77, 2305–2321. 10.3758/s13414-015-0927-0 26018643

[B16] Heinze H. J. Mangun G. R. (1995). Electrophysiological signs of sustained and transient attention to spatial locations. Neuropsychologia 33, 889–908. 10.1016/0028-3932(95)00023-v 7477815

[B17] Hickey C. Di Lollo V. McDonald J. J. (2009). Electrophysiological indices of target and distractor processing in visual search. J. Cogn. Neurosci. 21, 760–775. 10.1162/jocn.2009.21039 18564048

[B18] Hilimire M. R. Hickey C. Corballis P. M. (2012). Target resolution in visual search involves the direct suppression of distractors: evidence from electrophysiology. Psychophysiology 49, 504–509. 10.1111/j.1469-8986.2011.01326.x 22176697

[B19] Ikkai A. McCollough A. W. Vogel E. K. (2010). Contralateral delay activity provides a neural measure of the number of representations in visual working memory. J. Neurophysiol. 103, 1963–1968. 10.1152/jn.00978.2009 20147415 PMC2853266

[B20] Ishihara S. (1991). Ishihara’s Tests for Colour-Blindness (Tokyo, Japan: Kanehara Co.

[B21] Jolicoeur P. Brisson B. Robitaille N. (2008). Dissociation of the N2pc and sustained posterior contralateral negativity in a choice response task. Brain Res. 1215, 160–172. 10.1016/j.brainres.2008.03.059 18482718

[B22] Kiss M. Grubert A. Eimer M. (2013). Top-down task sets for combined features: behavioral and electrophysiological evidence for two stages in attentional object selection. Atten. Percept. Psychophys. 75, 216–228. 10.3758/s13414-012-0391-z 23143916

[B23] Kiss M. Grubert A. Petersen A. Eimer M. (2012). Attentional capture by salient distractors during visual search is determined by temporal task demands. J. Cogn. Neurosci. 24, 749–759. 10.1162/jocn_a_00127 21861683

[B24] Lamy D. Egeth H. E. (2003). Attentional capture in singleton-detection and feature-search modes. J. Exp. Psychol. Hum. Percept. Perform. 29, 1003–1020. 10.1037/0096-1523.29.5.1003 14585019

[B25] Leblanc E. Prime D. J. Jolicoeur P. (2008). Tracking the location of visuospatial attention in a contingent capture paradigm. J. Cogn. Neurosci. 20, 657–671. 10.1162/jocn.2008.20051 18052780

[B26] Liao H. I. Yeh S. L. (2013). Capturing attention is not that simple: different mechanisms for stimulus-driven and contingent capture. Atten. Percept. Psychophys. 75, 1703–1714. 10.3758/s13414-013-0537-7 24037596

[B27] Lien M. C. Gemperle A. Ruthruff E. (2011). Aging and involuntary attention capture: electrophysiological evidence for preserved attentional control with advanced age. Psychol. Aging 26, 188–202. 10.1037/a0021073 20973601

[B28] Lien M. C. Ruthruff E. Goodin Z. Remington R. W. (2008). Contingent attentional capture by top-down control settings: converging evidence from event-related potentials. J. Exp. Psychol. Hum. Percept. Perform. 34, 509–530. 10.1037/0096-1523.34.3.509 18505320

[B29] Lopez-Calderon J. Luck S. J. (2014). ERPLAB: an open-source toolbox for the analysis of event-related potentials. Front. Hum. Neurosci. 8:213. 10.3389/fnhum.2014.00213 24782741 PMC3995046

[B30] Lorenzo-Lopez L. Amenedo E. Cadaveira F. (2008). Feature processing during visual search in normal aging: electrophysiological evidence. Neurobiol. Aging 29, 1101–1110. 10.1016/j.neurobiolaging.2007.02.007 17346855

[B31] Luck S. J. Fuller R. L. Braun E. L. Robinson B. Summerfelt A. Gold J. M. (2006). The speed of visual attention in schizophrenia: electrophysiological and behavioral evidence. Schizophr. Res. 85, 174–195. 10.1016/j.schres.2006.03.040 16713184

[B32] Luck S. J. Hillyard S. A. (1994). Spatial filtering during visual search: evidence from human electrophysiology. J. Exp. Psychol. Hum. Percept. Perform. 20, 1000–1014. 10.1037/0096-1523.20.5.1000 7964526

[B33] Mazza V. Turatto M. Umilta C. Eimer M. (2007). Attentional selection and identification of visual objects are reflected by distinct electrophysiological responses. Exp. Brain Res. 181, 531–536. 10.1007/s00221-007-1002-4 17602216 PMC2258005

[B34] Mognon A. Jovicich J. Bruzzone L. Buiatti M. (2010). ADJUST: An automatic EEG artifact detector based on the joint use of spatial and temporal features. Psychophysiology 48, 229–240. 10.1111/j.1469-8986.2010.01061.x 20636297

[B56] Pivik R. T. Broughton R. J. Coppola R. Davidson R. J. Fox N. Nuwer M. R. (1993). Guidelines for the recording and quantitative analysis of electroencephalographic activity in research contexts. Psychophysiology 30, 547–558. 10.1111/j.1469-8986.1993.tb02081.x 8248447

[B35] Remington R. W. Folk C. L. (2001). A dissociation between attention and selection. Psychol. Sci. 12, 511–515. 10.1111/1467-9280.00394 11760140

[B36] Remington R. W. Folk C. L. McLean J. P. (2001). Contingent attentional capture or delayed allocation of attention? Percept. Psychophys. 63, 298–307. 10.3758/bf03194470 11281104

[B37] Reynolds J. H. Chelazzi L. (2004). Attentional modulation of visual processing. Annu. Rev. Neurosci. 27, 611–647. 10.1038/382539a0 15217345

[B38] Sawaki R. Geng J. J. Luck S. J. (2012). A common neural mechanism for preventing and terminating the allocation of attention. J. Neurosci. 32, 10725–10736. 10.1523/jneurosci.1864-12.2012 22855820 PMC3488698

[B39] Sawaki R. Luck S. J. (2010). Capture versus suppression of attention by salient singletons: electrophysiological evidence for an automatic attend-to-me signal. Atten. Percept. Psychophys. 72, 1455–1470. 10.3758/app.72.6.1455 20675793 PMC3705921

[B40] Sawaki R. Luck S. J. (2011). Active suppression of distractors that match the contents of visual working memory. Vis. Cogn. 19, 956–972. 10.1080/13506285.2011.603709 22053147 PMC3204803

[B41] Sawaki R. Luck S. J. (2013). Active suppression after involuntary capture of attention. Psychon. Bull. Rev. 20, 296–301. 10.3758/s13423-012-0353-4 23254574 PMC3845459

[B42] Sawaki R. Luck S. J. Raymond J. E. (2015). How attention changes in response to incentives. J. Cogn. Neurosci. 27, 2229–2239. 10.1162/jocn_a_00847 26151604 PMC4589447

[B43] Schneider D. Beste C. Wascher E. (2012). On the time course of bottom-up and top-down processes in selective visual attention: an EEG study. Psychophysiology 49, 1492–1503. 10.1111/j.1469-8986.2012.01462.x 22978270

[B44] Schneider D. Hoffmann S. Wascher E. (2014). Sustained posterior contralateral activity indicates re-entrant target processing in visual change detection: an EEG study. Front. Hum. Neurosci. 8:247. 10.3389/fnhum.2014.00247 24860467 PMC4017132

[B45] Schneider D. Mertes C. Wascher E. (2015). On the fate of non-cued mental representations in visuo-spatial working memory: Evidence by a retro-cuing paradigm. Behav. Brain Res. 293, 114–124. 10.1016/j.bbr.2015.07.034 26196953

[B46] Schneider D. Mertes C. Wascher E. (2016). The time course of visuo-spatial working memory updating revealed by a retro-cuing paradigm. Sci. Rep. 6: 21442. 10.1038/srep21442 26869057 PMC4751472

[B47] Schneider D. Wascher E. (2013). Mechanisms of target localization in visual change detection: an interplay of gating and filtering. Behav. Brain Res. 256, 311–319. 10.1016/j.bbr.2013.08.046 24001756

[B48] Theeuwes J. (2010). Top-down and bottom-up control of visual selection. Acta Psychol. (Amst) 135, 77–99. 10.1016/j.actpsy.2010.02.006 20507828

[B49] Theeuwes J. Atchley P. Kramer A. F. (2000). “On the time course of top-down and bottom-up control of visual attention, ” in Attention Performance, Vol. 2, ed. Driver S. M. J. (Cambridge, MA: MIT Press), 105–125.

[B50] Töllner T. Conci M. Rusch T. Müller H. J. (2013). Selective manipulation of target identification demands in visual search: the role of stimulus contrast in CDA activations. J. Vis. 13:23. 10.1167/13.3.23 23912067

[B51] Töllner T. Zehetleitner M. Gramann K. Müller H. J. (2010). Top-down weighting of visual dimensions: behavioral and electrophysiological evidence. Vis. Res. 50, 1372–1381. 10.1016/j.visres.2009.11.009 19925821

[B52] Verleger R. Koerbs A. Graf J. Smigasiewicz K. Schroll H. Hamker F. H. (2014). Patients with Parkinsons disease are less affected than healthy persons by relevant response-unrelated features in visual search. Neuropsychologia 62, 38–47. 10.1016/j.neuropsychologia.2014.07.004 25038550

[B53] Vogel E. K. Machizawa M. G. (2004). Neural activity predicts individual differences in visual working memory capacity. Nature 428, 748–751. 10.1038/nature02447 15085132

[B54] Wascher E. Wauschkuhn B. (1996). The interaction of stimulus- and response-related processes measured by event-related lateralizations of the EEG. Electroencephalogr. Clin. Neurophysiol. 99, 149–162. 10.1016/0013-4694(96)95602-3 8761051

[B55] Wiegand I. Töllner T. Dyrholm M. Müller H. J. Bundesen C. Finke K. (2014). Neural correlates of age-related decline and compensation in visual attention capacity. Neurobiol. Aging 35, 2161–2173. 10.1016/j.neurobiolaging.2014.02.023 24684790

